# Mental health training for primary health care workers and implication for success of integration of mental health into primary care: evaluation of effect on knowledge, attitude and practices (KAP)

**DOI:** 10.1186/s13033-017-0169-8

**Published:** 2017-10-12

**Authors:** Getinet Ayano, Dawit Assefa, Kibrom Haile, Asrat Chaka, Kelemua Haile, Melat Solomon, Kalkidan Yohannis, Akilew awoke, Kemal Jemal

**Affiliations:** 1 Research and Training Department, Amanuel Mental Specialized Hospital, PO Box: 1971, Addis Ababa, Ethiopia; 2 School of Public Health, University of Queensland, Brisbane, Australia; 3 Academic and Research, College of Health Science, Salale University, Fitche, Ethiopia

**Keywords:** Primary health care workers, Mental disorders, Mental health care, Integrated care, Primary health care, KAP, Quasi-experimental study

## Abstract

**Background:**

Mental disorders are always remained a neglected public health problems in low and middle-income countries (LMICs), most people with mental disorders never receive effective care and there is a large treatment gap. In order to solve the problem integration of mental health into primary health care is recommended and in Ethiopia implementation of the scale of mental health services at primary health care level was started in 2014. For the success of the integration of mental health into primary health care, primary care health professionals are the key personnel who are responsible for the management of mental, neurologic and substance use disorders. However, proper training and education of primary care health professionals is mandatory for an optimal performance and success of integration. This interventional study was conducted to assess the effectiveness of mental health training course for scale up of mental health services at primary health care level in Ethiopia.

**Methods:**

This quasi-experimental pre- and post-study design was conducted in Ethiopia from October to December 2016 using quantitative data collection methods. A total of 94 primary health care professionals were included in the study. The intervention was conducted by psychiatry professionals using standardized World Health Organization (WHO) Mental Health Gap Action Programme (mhGAP) guide prepared for scaling up of mental health care through integration into primary health care (PHC) and general medical services. Pre- and post intervention assessment was done for knowledge, attitude and practice (KAP); and statistically analyzed. A paired sample *t* test with p values was performed to test the differences between the pre- and post-test. In additions mean and standard deviation of the responses were calculated. Overall the response rate was 100% at the end of the intervention.

**Results:**

The study resulted in a significant improvement in knowledge, attitude and practice (KAP) of PHC workers about all the four mental, neurologic and substance use disorders during the post intervention survey (p < 0.05). Post intervention the knowledge of health professionals increased by 53.19% for psychosis, 42.56% for depression, 19.25% for epilepsy and 54.22% for alcohol use disorders. Similarly, post intervention attitude increased by 55.32% for psychosis, 40.42% for depression, 36.17% for epilepsy and 43.6% for alcohol use disorders. In addition, post intervention case identification rate increased by 62.78% for psychosis, 55.46% for depression, 21.35% for epilepsy and 41.49% for alcohol use disorders with significant p value (p < 0.05).

**Conclusions:**

The study results suggest that mental health training could be an effective intervention for improving knowledge, attitudes, and practices among primary health care professionals regarding mental, neurologic and substance use disorders. Training is a prerequisite and vital to enhance the knowledge, attitude, and practice of primary care professionals which plays a significant role for the easy success of integrated care and treatment of mental, neurologic and substance use disorders into the existing general health care services.

## Background

Mental, neurological and substance use (MNS) disorders constitute a huge global burden of disease, and there is a large treatment gap, particularly in low- and middle-income countries (LMICs) and they are major contributors to morbidity and premature mortality [1]. MNS disorders alone contribute 10.4% of the total global burden of disease, mostly accounted for by depression and other common mental disorders, alcohol and substance-use disorders, psychoses and epilepsy [2]. In Ethiopia, schizophrenia and depression are ranked in the top ten contributors to the total burden of disease in terms of DALYs [3]. In LMICs, such as Ethiopia mental disorders which are not considered as life-threatening problems are not given attention for a long time [4, 5]. As a result, mental health services are not given due priority and the needs of people for mental health care are not met [6, 7]. In Ethiopia, only 10% of people with severe mental disorders ever receive effective care [4]. Untreated mental disorders lead to disability, a substantial personal burden for affected individuals and their families, poor quality of life, hum rights abuses, stigma and discrimination, poverty, decreased productivity, suffering, poor physical health and premature mortality [8–12].

Despite the high morbidity and premature mortality due to mental disorders in LMICs, which are often comorbid with physical diseases, there is a scarcity of mental health specialists [1, 12, 13]. In order to address the problems and support huge neglect of people with mental disorders, World Health Organization (WHO) launched the Mental Health Gap Action Programme (mhGAP) for scaling up of mental health care through integration into primary health care (PHC) and general medical services [1]. The main aim of the program is treating MNS disorders at PHC level (non-specialized setting) by trained non-specialized professionals [14–17].

Scaling up of mental health care through integration into PHC and general medical services in Ethiopia was implemented in 2014 [13, 17]. The objectives of scaling up mental health care includes: an increase in the number of people receiving services (coverage); an increase in the range of services offered; services that are built on a scientific evidence base, usually with a service model that has been shown to be effective in a similar context; services made sustainable through policy formulation, implementation, and financing (strengthening of health systems) [1].

One of the major challenges of successful integration of mental health into PHC is the lack of adequate knowledge, positive attitude, and skills for mental health service of primary health care professionals participating in care and treatment of peoples at primary health care levels [18]. This paper contributes to our understanding effects of mental health training on KAP of primary health care workers in Ethiopia setting. Such information is essential in order to met the training and support needs of PHC workers and contribute to the successful scale up of mental health care. The aim of the study was, therefore, to evaluate the effect mental health training on KAP of primary health workers in Ethiopia.

## Methods

### Research design and period

Quasi-experimental a single group pre-test/post-test design was used in order to evaluate the effect of training on knowledge, attitude and practice of health professionals working at primary health care level in Ethiopia from October to December 2016. We carried pre-intervention measurements of knowledge, attitude and practice among participates of the total sample and we assessed post training knowledge and attitude of the same participants after 5 days of training. Post training practice was assessed after 3-month work experience at the primary health care level by reviewing charts and records.

### Study setting and aims

The study is conducted in Ethiopia. In Ethiopia mhGAP scale upfor people suffering from MNS disorders was implemented in different regions in 2014. The main aim of mhGAP program is implementation and Scaling up care for MNS disorders in PHC facilities (non-specialized health-care settings) by non-specialized professionals (working at first- and second-level facilities) [17, 19]. Selected professionals from different primary health care levels will take 5 days training on selected mhGAP priority disorders. These trained professionals receive periodic supervisions and training on site of work from well-experienced psychiatry professionals [13, 17, 19]. In addition, they have mentoring programs including e-mentoring system where they consult psychiatry professionals by using phones, emails and other electronic communications systems [13, 17, 19]. This study aimed at the evaluation of effects of training KAPof health professions.

### Description of the training

The training programme is the result of cooperation between the Federal ministry of health of Ethiopia, Addis Ababa Health Bureau, and Amanuel Mental specialized Hospital. Training was given on four selected WHO mhGAP priority MNS disorders for scale up service in Ethiopia [17, 19]. Training was given for 5 days based on WHO mhGAP guide prepared for trainees [16] by trained mental health specialist (MSc. in mental health) and Psychiatrists. Training on each disorder includes theory supplemented by videos presentation prepared by WHO for training purpose for scale up services.

Here are the four selected WHO mhGAP priority disorders:

- Alcohol use disorders
- Depression
- Psychosis
- Epilepsy

The training was given in four rounds and 23, 24, 24 and 23 health professionals participated in the first, second, third and fourth round training respectively.

### Study population and sampling

We used non-probability sampling technique and all participant who came for the training were included in the study. A total of 94 primary health care professionals (44 health officers, 22 diploma nurses and 28 BSC nurses) selected from different primary health care levels were included in the study.

### Data collection

Data were collected using a self-completed questionnaire that contained three sections.

#### Vignette case identification

Questionnaires were asked about vignette case identification after Vignette descriptions of common priority MNS disorders such as psychosis, depression, epilepsy and alcohol use disorders both pre- and post training. WHO study design with case vignettes has also been used in a study Butajira to assess attitude about mental disorders [20], on Ethiopian medical students [21] and as part of the national mental health plan in the United Republic of Tanzania [22] and to assess perception and attitude of the community about mental disorders in Ethiopia [23, 24].

#### Clinical experience and pre service training

Self-report of years of clinical experience and whether or not the respondent had received pre-service training in mental health care and whether or not the patient had experience in diagnosis and treatment of common mental, neurologic and substance use disorders. In addition, cases identified and treated post training at primary health care level by trained primary health care professionals were collected by the chart review after 3 months of the training.

#### Knowledge and attitudes questionnaire

A structured questionnaire to investigate knowledge and attitudes of PHC workers towards mental illness was developed for the purpose of the study. The questionnaire drew on previous research in this area from LMIC settings [25–27]. Knowledge was assessed by 19 item knowledge questionnaire. The response was graded as 2 for correct answers and of 1 for incorrect answers. Those who score above the total mean score of 19 item knowledge questionnaire were considered as having good knowledge and those who score below the total mean score were considered as having poor knowledge. Attitude was assessed by 5 item knowledge questionnaire. The response was graded as; 1 disagree, 2 neutral and 3 agree Those who score above the total mean score of 5 item attitude questionnaire were considered as having favorable attitude and those who score below the total mean score were considered as having unfavorable attitude.

#### Questionnaires used to assess knowledge

1. Genetic exposure causes the disorder
2. Use of psychoactive substance causes the disorder
3. Neurochemical imbalance causes the disorder
4. Loss of loved one causes the disorder
5. Conflict in marriage causes the disorder
6. Academic failure causes the disorder
7. Divorce causes the disorder
8. Physical or sexual abuse causes the disorder
9. Unemployment causes the disorder
10. Work overload causes the disorder
11. Financial constraints cause the disorder
12. Evil eye causes the disorder
13. Evil spirit causes the disorder
14. Curse causes the disorder
15. Due to sins committed causes the disorder
16. Will of God causes the disorder
17. Magic causes the disorder
18. The condition is treatable
19. The condition has good outcome in most patients

#### Attitude questionnaires

1. The person treated in the same health center with the general patient. What about you?
2. Traditional healers are better in effectiveness than our medical care in treating this condition? What about you?
3. The person can marry and may bring children’s?
4. The person can be employed and able to work effectively.
5. The person can leave with others in society?

### Data processing and analyses

The data were cleaned before final analyses by looking at the distribution of the data, identifying outliers and checking back against the original data. Data analysis was carried out using SPSS version 23 for Windows. Most of the responses to the structured questionnaire were analyzed descriptively, as percentages or summary measures of central tendency. Sociodemographic and other factors were analyzed and reported by using words and table. A paired sample t test was performed to test the differences between the pre- and post-test. In additions mean and standard deviation of the responses were calculated.

### Ethical clearance

Ethical clearance was obtained from the research ethics committee of Amanuel mental specialized hospital research and training department. Informed, written consent was obtained from each study participant. The right to withdraw from the research process at any point in time was respected. Privacy and strict confidentiality were maintained during the interview process.

## Results

### Socio demographic and other characteristics of respondents

A total of 94 participants were included in the study which makes the response rate 100%. The mean age of the respondents was 27.80 (± standard deviation = 11.70) years.

Among total participants, more than two-third of them 66 (70.21%) were females, 34 (36.14%) were married, 44 (46.81%) were between the ages of 25–30 years.

More than one-third of the study participants 44 (46.81%) were health officers, the majority of the participants, 70 (74.47%) were Orthodox Christians and around forty-two participants 44.68% were Oromo by ethnicity. Majority of participants, 72 (76.60%) had taken psychiatry courses during under graduate training and more than half of them had 4–7 years of experience (Table 1).

**Table 1:** Sociodemographicand other characters tics of health professionals participated in the study in Ethiopia, October to December, 2016 (n = 94)

Characteristics	Frequency	Percentage
Gender		
Male	28	29.79
Female	66	70.21
Age		
Less than or equal to 25	29	30.85
25–30	44	46.81
Greater than 30	21	22.34
Marital status		
Single	40	42.55
Married	34	36.17
Divorced	12	12.76
Separated/widowed	8	8.52
Ethnicity		
Amhara	24	25.53
Oromo	42	44.68
Tigray	15	15.96
Gurage	8	8.52
Others	5	5.31
Religion		
Orthodox christian	70	74.47
Muslim	10	10.64
Protestant	14	14.89
Educational level		
Diploma nurse	22	23.40
Health officer	44	46.81
BSC nurse	28	29.79
Taken psychiatry course in undergraduate training		
Yes	72	76.60
No	22	23.40
Year of experience		
Less than 4	23	24.47
4–7	50	53.19
Greater than 7	21	22.34

### Knowledge about mental, neurologic and substance use disorders

The respondent’s knowledge about different mental, neurologic and substance use disorders included in scale up program in Ethiopia is presented in Table 2. Pre-and post-training evaluation indicated that post-intervention proportion of the participant’s knowledge about mental, neurologic and substance use disorders showed significant improvement. Post intervention knowledge of the participants about psychosis increased from 34.04 to 87.23% (p = 000). Similarly, greatest improvement was observed after training on participant’s knowledge about depression, epilepsy, and alcohol use disorders (Table 2).

**Table 2:** Percentage distribution of knowledge of health professionals participated in the study, pre- and post training in Ethiopia, October to December, 2016 (n = 94)

Cases	Pre training				Post training				p (2 tailed)
	F	P	M	SD	F	P	M	SD	
Psychosis									0.000
Good	32	34.04	24.35	8.48	82	87.23	34.07	7.60	
Poor	62	65.96			12	12.77			
Depression									0.001
Good	45	47.87	26.06	8.99	85	90.43	34.68	7.09	
Poor	49	52.13			9	9.57			
Epilepsy									0.043
Good	70	74.47	25.37	8.52	88	93.62	34.78	6.99	
Poor	24	25.53			6	6.38			
Alcohol use disorder									0.001
Good	36	40.42	24.76	8.67	89	94.68	35.30	6.46	
Poor	58	59.58			5	5.32			

*F* frequency, *P* percentage, *M* median, *SD* standard deviation, and Good knowledge those who score above the total mean score of 19 item knowledge questionnaire, poor knowledge those who score below the total mean score

### Attitude about mental, neurologic and substance use disorders

The participants showed significant increase post- intervention in proportion of favorable attitude about mental, neurologic and substance use disorders. Post- intervention majority of the participants 89.36% (p = 0.000) for psychosis, 95.74% (p = 0.001), for depression 89.36% (p = 0.002) for epilepsy and 90.42% (p = 0.001) for alcohol use disorders showed favorable attitude (Table 3).

**Table 3:** Percentage distribution of attitude of health professionals participated in the study, pre -and post training in Ethiopia, October to December, 2016

Cases	Pre training				Post training				P (2 tailed)
	F	P	M	SD	F	P	M	SD	
Psychosis									0.000
Favorable	32	34.04	9.15	4.55	84	89.36	14.08	2.50	
Unfavorable	62	65.96			10	10.67			
Depression									0.001
Good	52	55.32	10.46	4.57	90	95.74	13.38	1.99	
Poor	42	44.68			4	4.26			
Epilepsy									0.002
Favorable	50	53.19	9.82	4.56	84	89.36	13.91	2.82	
Unfavorable	44	46.81			10	10.67			
Alcohol use disorder									0.001
Favorable	44	46.81	9.27	4.52	85	90.42	14.15	2.53	
Unfavorable	50	53.19			9	9.58			

*F* frequency, *P* percentage, *M* median, *SD* standard deviation, and Favorable attitude those who score above the total mean score of 5 item attitude questionnaire, unfavorable attitude those who score below the total mean score

### Practice of mental, neurologic and substance use disorders

Regarding practice of mental, neurologic and substance use (MNS) disorders, post- intervention the participant’s case detection and treatment showed significant improvement, which was assessed after three-month practice at primary health care level. Post intervention case identification of the participants increased from 9.53 to 20.84% (p = 001) for psychosis, and 15.87–18.75% (p = 0.001) for depression. Similarly, post- intervention case identification of the participants showed significant improvement for epilepsy and alcohol use disorders (Table 4).

**Table 4:** Percentage distribution of mental, neurologic and substance use disorders diagnosedand treated by health professionals participated in the study, pre and 3 month post training at primary health care in Ethiopia, October to December, 2016(n = 94)

Cases	Pre training		Post training (3 month experience)		P value
	Frequency	Percentage	Frequency	Percentage	
Psychosis	6	9.53	60	20.83	0.001
Depression	10	15.87	54	18.75	0.002
Epilepsy	32	50.79	134	46.53	0.014
Alcohol use disorder	5	7.94	16	5.56	0.027
Others	10	15.87	24	8.33	0.047
Total	63	100%	288	100%	

### Detection of vignette cases for mental, neurologic and substance use disorders

The respondent’s vignette case identification for different mental, neurologic and substance use disorders included in scale up program in Ethiopia is presented in Table 5. Pre and post training evaluation indicated that vignette case identification of the trainees about mental, neurologic and substance use disorders showed significant improvement after the training. The vignette case identification of trainees increased from 31.92 to 94.68% (p = 000) for psychosis, 34.04–90% (p = 001) for depression, 75.45–96.80% (p = 003), for epilepsy and 53.19–94.68% (p = 002) for alcohol use disorder (Table 5).

**Table 5:** Percentage distribution of vignette case identification by health professionals participated in the study, pre and post training in Ethiopia, October to December, 2016(n = 94)

Cases	Pre training		Post training		P value
	Frequency	Percentage	Frequency	Percentage	
Psychosis	30	31.92	89	94.68	0.000
Depression	32	34.04	88	90	0.001
Epilepsy	70	75.45	91	96.80	0.003
Alcohol use disorder	50	53.19	89	94.68	0.002

## Discussion

This a quasi-experimental pre- post-study is one its own kind of research which has meticulously dealt with PHC workers KAP towards MNS disorders and implication for the success of the integration of mental health into primary health care. The purpose of this study was to evaluate whether or not receipt of a training package specifically designed for integration of mental health care into primary health care improved in KAP related to common priority MNS disorders.

This study revealed that the pre training knowledge, attitude and practice of primary health care professionals about common priority mental, neurologic and substance use disorders is relatively low, which supports the finding that majority of people with mental, neurologic and substance use disorders don not receive adequate treatment and care in low and middle income(LMICs) countries due to lack of attention, problems related to of awareness and negative attitude by health care professionals [1, 17, 18].

Consistent with previous research [17, 18], primary health care workers who had taken the training improved in knowledge, attitude and practice related to common mental, neurologic and substance use disorders selected and implemented for scale and integration of mental health services into primary health care [17]. This findings indicated that training has huge impact and it’s vital for success of integrated treatment of mental, neurologic and substance use disorders with the existing general health care services.

According to the current study the participants showed significant increase post- intervention in proportion of knowledge about mental, neurologic and substance use. Pre- and post-training evaluation indicated that post-intervention proportion of the participant’s knowledge about mental, neurologic and substance use disorders showed significant improvement. Post intervention knowledge of the participants about psychosis increased from 34.04 to 87.23% (p = 000). Similarly, greatest improvement was observed after training on participant’s knowledge about depression, epilepsy and alcohol use disorders. This result indicates that training has significant effect on knowledge primary health care workers related to mental, neurologic and substance use disorders which is vital for success of integrated services. This findings are in line with other studies done in Nigeria [18] and other countries [28, 29].

This study demonstrated that training has significant effect on attitude of primary health care workers about mental, neurologic and substance use disorders. The effect is higher for psychosis followed depression and alcohol use disorders. Post-intervention the majority of the participants, 89.36% (p = 0.000) for psychosis, 95.74% (p = 0.001), for depression 89.36% (p = 0.002) for epilepsy and 90.42% (p = 0.001) for alcohol use disorders showed favorable attitude. This findings are in agreement with other studies done in Nigeria [18] and other countries [28, 29].

We found a change in the practice of primary health care professionals about mental, neurologic and substance use disorders after the training. This findings suggests that effectiveness of mental health training on practice of primary health care workers and continues training and education is vital for success of mental health integration into general health care services. Post- intervention the participants case detection and treatment showed significant improvement, which was assessed after three-month practice at primary health care level. Post intervention case identification of the participants increased from 9.53% to 20.84% (p = 001) for psychosis, and 15.87 to 18.75% (p = 0.001) for depression. Similarly, post- intervention case identification of the participants showed significant improvement for epilepsy and alcohol use disorders. This findings are in agreement with other studies done in Nigeria [18] and other countries [28, 29].

This study also indicated that there is statically significant difference in vignette case identification of primary health care workers pre- and post-training. The intervention had a large impact on case identification of primary health care workers, which is essential for the success of the integration of mental health into primary health care services. The vignette case identification of trainees increased from 31.92 to 94.68% (p = 000) for psychosis, 34.04 to 90% (p = 001) for depression, 75.45 to 96.80% (p = 003), for epilepsy and 53.19 to 94.68% (p = 002) for alcohol use disorder. This findings are in agreement with other studies done in Nigeria [18] and other countries [28, 29].

## Conclusion

The study resulted in a significant improvement in KAP of PHC workers about all the four mental, neurologic and substance use disorders during the post intervention survey. PHC workers showed significant increase post intervention in proportion of knowledge, attitude and practice related to common mental, neurologic and substance use disorders selected and implemented for scale and integration of mental health services into primary health care in Ethiopia. Training is a prerequisite and vital to enhance the knowledge, attitude, and practice of primary care professionals which plays a significant role for the easy success of integrated care and treatment of mental, neurologic and substance use disorders into the existing general health care services.

The mail limitations of this study was failure discuss and compare the results with other previous studies due to lack adequate studies in the area.

## References

[1] World Health Organization (2008). Mental Health Gap Action Programme (mhGAP): scaling up care for mental, neurological, and substance use disorders.

[2] Murray CJL, Vos T, Lozano R, Naghavi M, Flaxman AD, Michaud C, Ezzati M, Shibuya K, Salomon JA, Abdalla S, Aboyans V, Abraham J, Ackerman I, Aggarwal R, Ahn SY, Ali MK, Alvarado M, Anderson HR, Anderson LM, Andrews KG, Atkinson C, Baddour LM, Bahalim AN, Barker-Collo S, Barrero LH, Bartels DH, Basáñez M-G, Baxter A, Bell ML, Benjamin EJ (2012). Disability-adjusted life years (DALYs) for 291 diseases and injuries in 21 regions, 1990–2010: a systematic analysis for the Global Burden of Disease Study 2010. Lancet.

[3] Abdulahi H, Haile-Mariam D, Kebede D (2001). Burden of disease analysis in rural Ethiopia. Ethiop Med J.

[4] World Bank (1993). World development report: investing in mental health.

[5] Desjardins R, Eisenburg L (1995). World mental health: problems and priorities in low income countries.

[6] Kohn R, Saxena S, Levav I, Saraceno B (2004). He treatment gap in mental health care. Bull World Health Organ.

[7] Wang PS, Angermeyer M, Borges G, Bruffaerts R, Chiu WT (2007). Delay and failure in treatment seeking after first onset of mental health disorders in the World Health Organization’s World Mental Health Survey Initiative. World Psychiatry.

[8] Alem A, Kebede D, Fekadu A, Shibre T, Fekadu D (2009). Clinical course and outcome of schizophrenia in a predominantly treatment-naive cohort in rural Ethiopia. Schizophr Bull.

[9] Kebede D, Alem A, Shibre T, Negash A, Deyassa N (2005). Shortterm symptomatic and functional outcomes of schizophrenia in Butajira, Ethiopia. Schizophr Res.

[10] HornLcroi G, Brohan E, Rose D, Sartorius N, Leese M, INDIGO Study Group (2009). Global pattern of experienced and anticipated discrimination against people with schizophrenia: a cross-sectional survey. Lancet.

[11] Lund C, Breen A, Flisher AJ, Kakuma R, Corrigall J (2010). Poverty and common mental disorders in low and middle income countries: a systematic review. SocSci Med.

[12] Teferra S, Shibre T, Fekadu A, Medhin G, Wakwoya A (2011). Fiveyear mortality in a cohort of people with schizophrenia in Ethiopia. BMC Psychiatry.

[13] Ayano G (2016). Primary Mental Health Care Services in Ethiopia: experiences, Opportunities and Challenges from East African Country. J Neuropsychopharmacol Ment Health.

[14] Barbui C, Dua T, van Ommeren M, Yasamy MT, Fleischmann A, Clark N, Thornicroft G, Hill S, Saxena S (2010). Challenges in developing evidence-based recommendations using the GRADE approach: the case of mental, neurological, and substance use disorders. PLoS Med.

[15] Dua T, Barbui C, Clark N, Fleischmann A, Poznyak V (2011). Evidence-based guidelines for mental, neurological, and substance use disorders in low- and middle-income countries: summary of WHO recommendations. PLoS Med.

[16] World Health Organization (2010). Mental Health Gap Action Programme Implementation Guide (mhGAP-IG) for mental, neurological and substance use disorders in non-specialized health settings.

[17] Ayano G, Assefa D, Haile K, Bekana L (2016). Experiences, strengths and challenges ofintegration of mental health into primary care in Ethiopia. Fam Med Med Sci Res.

[18] AdebowaleT Umukoro LU, Richard Gater R, Akinhanmi A, Ogunlesi A (2014). Evaluation of a Mental Health Training Course forPrimary Health Care Workers in Ogun State, South West. Nigeria J Psychiatry.

[19] mhGAP in Ethiopia: proof of concept 2013.

[20] Alem A, Jacobson Araya M, Kebede D, Kullgren GH (1999). How are mental disorders seen and where help sought in rural Ethiopian community? A key informant study in Butajira, Ethiopia. Acta Psychiatr Scand.

[21] Khandelwal S, Workneh F (1986). Perception of mental illness by medical students. Ind J Psychol Med.

[22] Whyte SR (1991). Attitudes towards mental health problems in Tanzania. Acta Pscychiatry.

[23] Duko B, Ayano G, Agidew M (2016). Perception, Attitude and Correlates of Alcoholism and Epilepsy among Residents of Hawassa City, South Ethiopia, Cross Sectional Study. Afr J Psychiatry (South Afr).

[24] Ayano G (2015). MelkamuAgidew, BereketDuko, HaregwoinMulat, MelkamuAlemayew. (2016) Perception, Attitude and Associated Factors on Schizophrenia and Depression among Residents of Hawassa City, South Ethiopia, Cross Sectional Study. American. J Psychiatry Neurosci.

[25] Deribew A, Tesfaye M. Assessment of knowledge, attitude and practice of nursing staff towards mental health problems in Jimma zone, south western Ethiopia. Ethiop J Health Sci. 2005, 15.

[26] Ignacio LL, de Arango MV, Baltazar J, Busnello ED, Climent CE, Elhakim A, Farb M, Guèye M, Harding TW, Ibrahim HH, Murthy RS, Wig NN (1983). Knowledge and attitudes of primary health care personnel concerning mental health problems in developing countries. Am J Public Health.

[27] Shyangwa PM, Singh S, Khandelwal SK (2003). Knowledge and attitude about mental illness among nursing staff. J Nepal Med Assoc.

[28] Cohen A. The effectiveness of mental health services in primary care: The view from the developing world. Publication WHO/MQ/MPS/01.1.Department of Mental Health and Substance Dependence 2001, Geneva: WHO.

[29] Hodges B, Inch C, Silver I (2001). Improving the psychiatric knowledge, skills, and attitudes of primary care physicians, 1950–2000: a review. Am J Psychiatry.

