# Correction to: Mental health training for primary health care workers and implication for success of integration of mental health into primary care: evaluation of effect on knowledge, attitude and practices (KAP)

**DOI:** 10.1186/s13033-017-0175-x

**Published:** 2017-11-10

**Authors:** Getinet Ayano, Dawit Assefa, Kibrom Haile, Asrat Chaka, Kelemua Haile, Melat Solomon, Kalkidan Yohannis, Akilew Awoke Adane, Kemal Jemal

**Affiliations:** 1 Research and Training Department, Amanuel Mental Specialized Hospital, PO Box: 1971, Addis Ababa, Ethiopia; 2 School of Public Health, University of Queensland, Brisbane, Australia; 3 Academic and Research, College of Health Science, Salale University, Fitche, Ethiopia

## Correction to: Int J Ment Health Syst (2017) 11:63 10.1186/s13033-017-0169-8

Following publication of the original article [1], the authors identified two errors in the author details, and requested the following changes:

1. That the corresponding author’s email address be changed to babiget2015@gmail.com.
2. That the name of co-author Akilew Awoke be changed to Akilew Awoke Adane.

With regard to (2), the original article has been corrected.
